# Associations between depressive symptoms and frequency, intensity, duration, and style of walking in survivors of the Great East Japan Earthquake

**DOI:** 10.1002/pcn5.70178

**Published:** 2025-09-14

**Authors:** Yusuke Utsumi, Moe Seto, Hitomi Usukura, Yumiko Hamaie, Atsushi Sakuma, Kazuho Tomimoto, Hiroshi Komatsu, Saya Kikuchi, Yumi Sugawara, Shinichi Kuriyama, Naoki Nakaya, Atsushi Hozawa, Yasuto Kunii, Hiroaki Tomita

**Affiliations:** ^1^ Department of Psychiatry Tohoku University Hospital Sendai Japan; ^2^ Department of Psychiatry Graduate School of Medicine, Tohoku University Sendai Japan; ^3^ Department of Disaster Psychiatry International Research Institute of Disaster Science, Tohoku University Sendai Japan; ^4^ Department of Human Science Faculty of Liberal Arts, Tohoku Gakuin University Sendai Japan; ^5^ Department of Public Health Graduate School of Medicine, Tohoku University Sendai Japan; ^6^ Department of Preventive Medicine and Epidemiology Tohoku Medical Megabank Organization, Tohoku University Sendai Japan; ^7^ Department of Disaster Public Health International Research Institute of Disaster Science, Tohoku University Sendai Japan

**Keywords:** health consciousness, mental Health, physical activity, post‐disaster communities, walking parameter

## Abstract

**Background:**

In post‐disaster communities, an association between decreased walking activity and depressive symptoms has been reported. This study aimed to identify the associations between the frequency, intensity, time, and type (or style) (FITT) of walking and depressive symptoms.

**Method:**

The 2018 survey of a cohort study was used to examine 924 individuals aged 20 years or older who were severely affected by the Great East Japan Earthquake. Participants were asked whether they walked intending to improve their health (health‐conscious walkers: *N* = 335) and were cautious about their walking parameters. Depressive symptoms were assessed using the Center for Epidemiologic Studies Depression Scale (CES‐D), and multivariate logistic regression analysis was used to evaluate the association between paying attention to FITT elements and depressive symptoms.

**Results:**

In health‐conscious walkers, the multivariate model showed that female (odds ratio [OR], 2.45; 95% confidence interval [CI], 1.24–4.84) and paying attention to posture during walking (OR, 0.41; 95% CI, 0.21–0.81) were significantly associated with depressive symptoms (CES‐D ≥ 16). In non‐health‐conscious walkers, evaluating multiple variables, including walking duration, showed that only a walking duration of less than 30 min per day (OR, 2.06; 95% CI, 1.19–3.56) was associated with depressive symptoms.

**Conclusions:**

The current study indicated that paying attention to posture during walking had a significant negative association with depressive symptoms, suggesting that paying attention to posture while walking may be beneficial for mental health well‐being. These findings may help improve the mental health of communities affected by a disaster through an intervention to promote regular walking.

## Graphical abstract

In a survey examining adult residents whose houses were devastated by the 2011 Great East Japan Earthquake, participants were asked whether they intended to improve their health (health‐conscious walkers) and were cautious about the frequency, intensity, time, and style during their walking. The multivariate model showed that paying attention to posture during walking had a significant negative association with depressive symptoms. The findings may help improve the mental health of communities affected by a disaster through an intervention to promote regular walking.

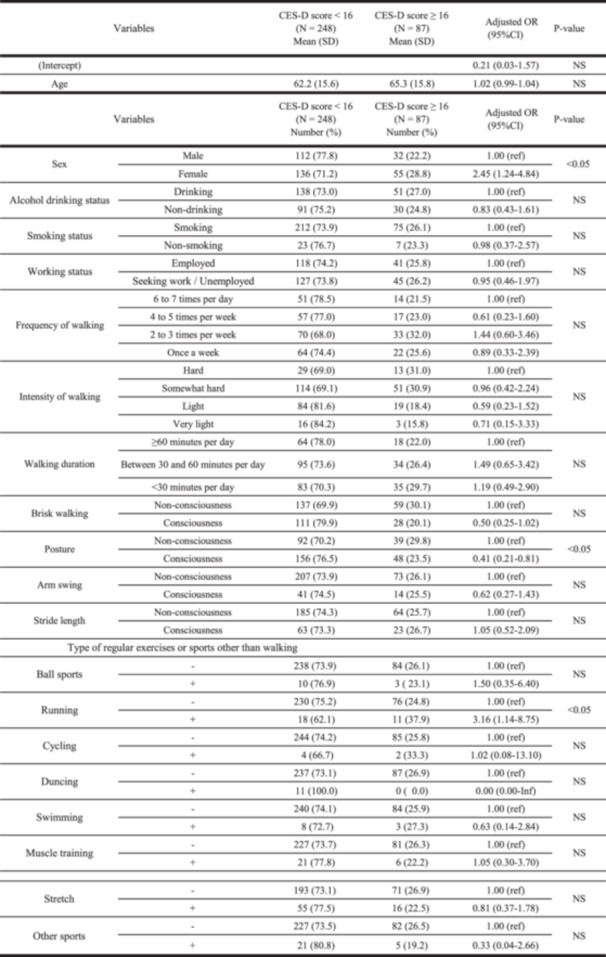

## BACKGROUND

In communities affected by a disaster, decreased physical activity and a subsequent increased risk of mental health problems have been reported.^1^, ^2^ For example, after the Great East Japan Earthquake (GEJE) that occurred on March 11, 2011, several studies reported that the physical activity level of evacuees living in temporary housing in the damaged area was lower than the national average in Japan.^3^, ^4^ Generally, physical activities,^5^, ^6^, ^7^, ^8^, ^9^, ^10^ including walking,^11^, ^12^, ^13^ are positively associated with mental health and negatively associated with depressive symptoms, regardless of age and sex. However, the relationship between physical activities and mental health condition has not been sufficiently examined in post‐disaster settings, though a disaster affects the lifestyles and mental health conditions of the affected communities.^14^, ^15^, ^16^, ^17^, ^18^, ^19^, ^20^, ^21^ A few studies have reported that decreased physical activity is significantly associated with depressive symptoms in elderly persons and children in post‐disaster settings.^22^, ^23^ A recent study further showed that, in people who walked to maintain good health conditions, depressive symptom scores as continuous variables were significantly different between subgroups with longer and shorter walking durations.^24^

Walking is among the most widely applicable exercises, even for residents in areas where a disaster damages facilities for exercise. It may be useful to evaluate the associations between walking habits and mental health condition in a post‐disaster setting to promote regular walking after a disaster. In addition, people with depressive symptoms may face considerable barriers to continuing regular physical activity, such as a lack of motivation or pessimism.^25^, ^26^ Motivation or positive expectations for the future to maintain health may be necessary to continue regular physical exercise and may therefore lead to improved mental health and well‐being.

It has been pointed out that walking habits include both walking as a means of transportation and walking for health.^27^ Moreover, the impact of walking on physical and mental health may differ depending on whether the walking is health‐oriented or not, and among health‐conscious walkers, longer walking duration has been associated with a greater reduction in depressive symptoms.^24^ Accordingly, people who walk with the motivation to maintain their health tend to be cautious about the manner of walking. In general, the manner of exercise consists of frequency (F), intensity (I), time or duration (T), and type or style (T), abbreviated as “FITT.” The FITT principle is recommended by the American College of Sports Medicine to maintain cardiorespiratory fitness, body composition, muscular strength, and endurance as a comprehensive exercise regimen. FITT is important in the rehabilitation domain, and exercise prescription using the FITT principle is reportedly effective for patients with stroke,^28^ cardiovascular disease,^29^ and chronic obstructive pulmonary disease.^30^ In recent years, exercise prescriptions using the FITT principle have also been recommended for mental health. Further, regarding an association between walking style and depressive symptoms was reported in some previous studies. Those studies reported that decreased walking speed, stride length, arm swing, and slumped posture during walking were associated with depressive symptoms.^31^, ^32^, ^33^

Our previous study, based on the health survey for a community affected by the GEJE conducted in 2017, showed that depressive symptom scores were significantly different between subgroups with longer and shorter walking durations among health‐conscious walkers.^24^ However, it remains unelucidated whether consciousness on manner of walking, such as FITT, is associated with depressive symptoms, while these information can be useful to improve mental health conditions of people affected by a disaster through intervening walking habits. Therefore, when we conducted consecutive survey for the same community in 2018, we asked how the affected residents were conscious about FITT and analyzed the associations between depression and the comprehensively evaluated walking manner from the viewpoint of FITT.

## METHODS

### Study design, setting, and participants

The present study was based on data collected in a series of annual health surveys conducted in Shichigahama town, located on the coast of Miyagi Prefecture, which was severely affected by the GEJE, between 2011 and 2020, as previously described. Also, detailed inclusion criteria of participants and the method of the survey can be referred in the previous study based on the seventh survey conducted in 2017.^24^ In brief, participants were recruited from all residents who lived in Shichigahama town at the onset of the GEJE and whose houses were majorly damaged based on the official property damage evaluation criteria. This study was conducted using data obtained through self‐administered questionnaires from the 8th survey conducted in 2018, in which we asked additional questions focused on walking‐related factors (duration, frequency, intensity, and type). The study protocol was approved by the ethics committee of the Tohoku University Graduate School of Medicine (2021‐1‐618).

**Figure 1:**
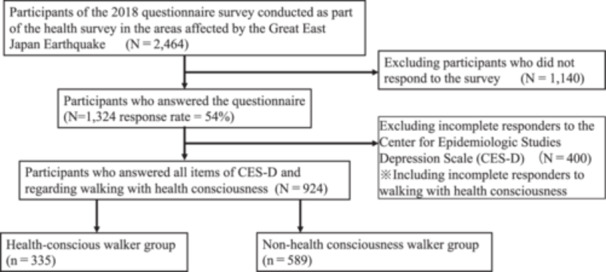
Flow of participants in the 2018 questionnaire survey and the present study.

Of the 2464 participants who met the inclusion criteria, 1324 responded (response rate = 54%). Of these, 924 completed the questionnaires used in the analyses. Furthermore, the 924 participants were divided into two groups: health‐conscious (*n* = 335) and non‐health‐conscious walkers (*n* = 589) (Figure 1).

### Questionnaires

The classification of health‐conscious and non‐health‐conscious walkers was determined by asking whether the purpose of walking is for health in the questionnaire, as previously described.^24^ Walking durations per day of the participants were evaluated in the same way as the previous the seventh survey conducted in 2017.^24^ In brief, walking duration was select from one of the following items”: (1) more than 60 min per day (≥60 min per day); (2) between 30 and 60 min per day; or (3) less than 30 min per day (<30 min per day) and whether walking to health or not (classified as “health‐conscious walkers” or “non‐health‐conscious walkers”).^24^ In the current study, based on the eighth survey conducted in 2018, the following four additional questions were newly added for the participants who answered that they were health‐conscious walkers. Non‐health‐conscious walkers are those who walk for purposes other than health (e.g., commuting or attending school). Therefore, we considered that it would be difficult for them to accurately respond to the FITT‐related questionnaires (as follows: questionnaires I, II, and III. I: The participants were asked, “How often do you walk?,” with the responses as follows: (1) 6–7 times per week; (2) 4–5 times per week; (3) 2–3 times per week; (4) once a week; and (5) 1–2 times per month. II: The participants were also asked, “How intensely do you walk?,” and the responses were based on Borg's rating of perceived exertion [(RPE): (1) very hard; (2) hard; (3) somewhat hard; (4) light; (5) very light; and (6) extremely light].^34^, ^35^ III: The participants were asked, “What do you pay attention to during your walk?,” and the responses were as follows: (1) brisk walking; (2) posture; (3) stride length; and (4) arm swing. IV: The participants were also asked “Do you engage in regular exercise or sports other than walking?.” When the answer was “Yes,” they were asked to select the types of exercise or sports from the following options: (1) stretching; (2) muscle training; (3) running (including jogging); (4) swimming; (5) cycling; (6) ball games; (7) dancing; and (8) others. Depressive symptoms were evaluated based on the Center for Epidemiologic Studies Depression Scale (CES‐D) as previously described.^24^, ^36^, ^37^ The information to be used as confounding factors, including drinking, smoking habitats were also evaluated using the questionnaire described in the previous publications.^14^, ^15^, ^16^, ^17^, ^18^, ^19^, ^20^, ^21^, ^24^

### Statistical analyses

Differences in the following factors between the health‐conscious and non‐health‐conscious walker groups were examined using the *χ* ^2^ test and *t*‐test: age, sex (male or female), number of family members, residential status (living in the same place as before the GEJE, living in a different place, not due to the disaster, post‐disaster publicly funded rental accommodation, houses of relatives or friends, prefabricated post‐disaster public housing, newly built houses in the relocated high‐land area, newly built houses outside of the relocated high‐land area, or others), economic status (extremely difficult, difficult, a little difficult, or fine), alcohol drinking (drinking or non‐drinking), smoking (smoking or non‐smoking), health status (good or difficult), working status (working or non‐working), walking duration (≥60 min per day, between 30 and 60 min per day, <30 min per day), and regular exercise or sports other than walking (stretching, muscle training, running, swimming, cycling, ball games, dancing, others). Residual analyses were done for the three categories of walking duration.

Frequency, intensity, time (duration), and type (style) of walking were assessed in health‐conscious walkers. Participants were asked to select one of five items regarding frequency of walking (six to seven times per day, four to five times per week, two to three times per week, once a week, and one to two times per month) and seven items regarding intensity of walking (extremely hard, very hard, hard, somewhat hard, light, very light, and extremely light). Regarding the frequency of walking, the selected items were integrated into four items (six to seven times per day, four to five times per week, two to three times per week, less than once a week). For intensity of walking, the selected items were integrated into four items (hard, somewhat hard, light, very light). Furthermore, participants were asked to select one of three categories regarding walking duration and four items regarding the style of walking (brisk walking, posture, stride length, and arm swing).

To evaluate the associations between FITT factors and depressive status (CES‐D scores ≥ 16) in health‐conscious walkers, multivariate logistic regression analyses were performed. In the multivariate model applied to health‐conscious walkers, the odds ratios (ORs) were adjusted for the following confounding factors: age, sex, alcohol drinking, smoking, working status, and regular exercise or sports. For non‐health‐conscious walkers, a multivariate model was used to evaluate the association between walking duration and depressive status (CES‐D scores ≥ 16), with the same confounding factors used for health‐conscious walkers. Furthermore, because a significant age difference was observed between health‐conscious and non‐health‐conscious walkers (as shown in Table 1), and since age is also associated with depressive symptoms,^39^ participants were stratified into three age groups: young (<40 years), middle‐aged (40–65 years), and older adults (≥65 years). This stratification aimed to address the potential confounding effect of age in the association between walking characteristics and depressive symptoms. Logistic regression analysis was then performed to evaluate the association between health‐conscious or non‐health‐conscious and depressive symptoms within each age group.

**Table 1:** Demographic information (number and proportion of participants with or without health‐consciousness).

Factor	Group	Health‐consciousness walkers (*N* = 335), mean (SD)	Non‐health‐ consciousness walkers (*N* = 589), mean (SD)	*p* value	
Age		63.0 (15.7)	53.2 (18.5)	<0.001	
The number of family members		3.7 (1.7)	3.9 (1.8)	NS	
CES‐D		12.2 (7.2)	12.0 (8.5)	NS	

Factor	Group	Health‐consciousness walkers (*N* = 335), Number (%)	Non‐health‐ consciousness walkers (*N* = 589) Number (%)	*p* value	Residual analysis, *p* value
Sex	Male	144 (35.3)	264 (64.7)	NS	
	Female	191 (37.0)	325 (63.0)		
Residential condition	Living in the same place as before the GEJE	98 (42.4)	133 (57.6)	NS	
	Living in a different place not due to the disaster	10 (21.3)	37 (78.7)		
	Houses of relatives or friends	7 (29.2)	17 (70.8)		
	Prefabricated post‐disaster public housing	27 (37.0)	46 (63.0)		
	Newly built houses in the relocated high‐land area	87 (37.5)	145 (62.5)		
	Newly built houses outside of the relocated high‐land area	66 (33.0)	134 (67.0)		
	Other	27 (32.1)	57 (67.9)		
Health status	≥Somewhat good	247 (35.5)	448 (64.5)	NS	
	≤Somewhat bad	79 (36.6)	137 (63.4)		
Alcohol drinking status	Drinking	121 (35.0)	225 (65.0)	NS	
	Non‐drinking	189 (36.2)	333 (63.8)		
Smoking status	Smoking	30 (19.0)	128 (81.0)	<0.001**	
	Non‐smoking	287 (39.9)	433 (60.1)		
Working status	Employed	159 (28.4)	401 (71.6)	<0.001**	
	Seeking work/Unemployed	172 (48.2)	185 (51.8)		
Perceived economic status	Extremely difficult	11 (22.0)	39 (78.0)	NS	
	Difficult	52 (38.2)	84 (61.8)		
	A little difficult	105 (37.1)	178 (62.9)		
	Fine	162 (36.4)	283 (63.6)		
Regular exercises or sports other than walking	Playing	185 (28.7)	459 (71.3)	<0.001	
	Non‐playing	150 (53.6)	130 (46.4)		
Walking duration	≥60 min per day	82 (28.5)	206 (71.5)	<0.001	<0.01
	Between 30 and 60 min per day	129 (45.3)	156 (54.7)		<0.001
	<30 min per day	118 (34.9)	220 (65.1)		NS
*Type of regular exercises or sports other than walking*					
Ball games	−	322 (36.2)	568 (63.8)	NS	
	+	13 (38.2)	21 (61.8)		
Running	−	306 (34.6)	578 (65.4)	<0.001	
	+	29 (72.5)	11 (27.5)		
Cycling	−	329 (35.9)	587 (64.1)	<0.05	
	+	6 (75.0)	2 (25.0)		
Duncing	−	324 (35.8)	582 (64.2)	<0.05	
	+	11 (61.1)	7 (38.9)		
Swimming	−	324 (35.7)	584 (64.3)	<0.01	
	+	11 (68.8)	5 (31.2)		
Muscle training	−	308 (35.6)	557 (64.4)	NS	
	+	27 (45.8)	32 (54.2)		
Stretching	−	264 (33.0)	537 (67.0)	<0.001	
	+	71 (57.7)	52 (42.3)		
Other sports	−	309 (35.3)	566 (64.7)	<0.05	
	+	26 (53.1)	23 (46.9)		

*Note*: The table summarizes the distribution of the participants by age, number of family members, the Center for Epidemiologic Studies Depression Scale (CES‐D), sex, residential status (living in the same place as before the GEJE, living in a different place for reasons other than the consequence of the GEJE, post‐disaster publicly funded rental accommodation, houses of relatives or friends, prefabricated post‐disaster public housing, newly built houses in the relocated high‐land area, newly built houses outside of the relocated high‐land area, others), health status (≥somewhat good, ≤somewhat bad), alcohol drinking status (drinking, non‐drinking), smoking status (smoking, non‐smoking), working status (employed, seeking work and unemployed), perceived economic status (extremely difficult, difficult, a little difficult, fine), regular exercise or sports other than walking (ball games, running, cycling, dancing, swimming, muscle training, stretching, others), and walking duration (≥60 min per day, between 30 and 60 min per day, <30 min per day). Differences in the above parameters between health‐conscious and non‐health‐conscious groups are shown using the *t*‐test and *χ* ^2^ test. Residual analysis was performed for walking duration.

Abbreviations: NS, not significant; SD, standard deviation.

Once significant associations between FITT factors and depressive status were identified, the differences in age, sex, alcohol drinking, smoking, work status, and regular exercise or sports between the subpopulations classified by the FITT factors were analyzed by the *χ* ^2^ test or the *t*‐test, as appropriate.

All statistical analyses were performed using EZR version 1.55.^38^ Significance was set at *p* value < 0.05.

## RESULTS

All participants (*N* = 924; 408 males and 516 females) completed all questions regarding walking habits and depressive symptoms. There was a significant difference in walking duration between health‐conscious and non‐health‐conscious walkers (*p* < 0.001). On residual analysis, in health‐conscious walkers, there was a significantly lower proportion of persons with walking duration >60 min per day (*p* < 0.005) and a significantly higher proportion with walking duration between 30 and 60 min per day (*p* < 0.001) compared with non‐health‐conscious walkers. There were significant differences between health‐conscious and non‐health‐conscious walkers in age (*p* < 0.001), smoking (*p* < 0.001), work status (*p* < 0.001), and involvement in running (*p* < 0.001), cycling (*p* < 0.05), dancing (*p* < 0.05), swimming (*p* < 0.01), stretching (*p* < 0.001), and other exercises/sports (*p* < 0.05), as summarized in Table 1.

On logistic regression analysis to investigate factors affecting depressed status (CES‐D ≥ 16) in health‐conscious walkers, depressed status was significantly associated with female (OR, 2.45; 95% CI, 1.24–4.84) and regular running (OR, 3.16; 95% CI, 1.14–8.75) and significantly negatively associated with paying attention to posture (OR, 0.41; 95% CI, 0.21–0.81) during walking (Table 2). Variance inflation factors (VIF) of the frequency of walking, intensity of walking, walking duration, and style of walking were less than 2.

**Table 2:** Logistic regression analysis of the effects of walking‐related factors on depressive symptoms in health‐conscious walkers.

Variables		CES‐D score < 16 (*N* = 248), mean (SD)	CES‐D score ≥ 16 (*N* = 87), mean (SD)	Adjusted OR (95% CI)	*p* value
(Intercept)				0.21 (0.03–1.57)	NS
Age		62.2 (15.6)	65.3 (15.8)	1.02 (0.99–1.04)	NS

Variables		CES‐D score < 16 (*N* = 248), Number (%)	CES‐D score ≥ 16 (*N* = 87), Number (%)	Adjusted OR (95% CI)	*p* value
Sex	Male	112 (77.8)	32 (22.2)	1.00 (ref)	<0.05
	Female	136 (71.2)	55 (28.8)	2.45 (1.24–4.84)	
Alcohol drinking status	Drinking	138 (73.0)	51 (27.0)	1.00 (ref)	NS
	Non‐drinking	91 (75.2)	30 (24.8)	0.83 (0.43–1.61)	
Smoking status	Smoking	212 (73.9)	75 (26.1)	1.00 (ref)	NS
	Non‐smoking	23 (76.7)	7 (23.3)	0.98 (0.37–2.57)	
Working status	Employed	118 (74.2)	41 (25.8)	1.00 (ref)	NS
	Seeking work/Unemployed	127 (73.8)	45 (26.2)	0.95 (0.46–1.97)	
Frequency of walking	6 to 7 times per day	51 (78.5)	14 (21.5)	1.00 (ref)	NS
	4 to 5 times per week	57 (77.0)	17 (23.0)	0.61 (0.23–1.60)	
	2 to 3 times per week	70 (68.0)	33 (32.0)	1.44 (0.60–3.46)	
	Once a week	64 (74.4)	22 (25.6)	0.89 (0.33‐2.39)	
Intensity of walking	Hard	29 (69.0)	13 (31.0)	1.00 (ref)	NS
	Somewhat hard	114 (69.1)	51 (30.9)	0.96 (0.42–2.24)	
	Light	84 (81.6)	19 (18.4)	0.59 (0.23–1.52)	
	Very light	16 (84.2)	3 (15.8)	0.71 (0.15–3.33)	
Walking duration	≥60 min per day	64 (78.0)	18 (22.0)	1.00 (ref)	NS
	Between 30 and 60 min per day	95 (73.6)	34 (26.4)	1.49 (0.65–3.42)	
	<30 min per day	83 (70.3)	35 (29.7)	1.19 (0.49–2.90)	
Brisk walking	Non‐consciousness	137 (69.9)	59 (30.1)	1.00 (ref)	NS
	Consciousness	111 (79.9)	28 (20.1)	0.50 (0.25–1.02)	
Posture	Non‐consciousness	92 (70.2)	39 (29.8)	1.00 (ref)	<0.05
	Consciousness	156 (76.5)	48 (23.5)	0.41 (0.21–0.81)	
Arm swing	Non‐consciousness	207 (73.9)	73 (26.1)	1.00 (ref)	NS
	Consciousness	41 (74.5)	14 (25.5)	0.62 (0.27–1.43)	
Stride length	Non‐consciousness	185 (74.3)	64 (25.7)	1.00 (ref)	NS
	Consciousness	63 (73.3)	23 (26.7)	1.05 (0.52–2.09)	
*Type of regular exercises or sports other than walking*					
Ball games	−	238 (73.9)	84 (26.1)	1.00 (ref)	NS
	+	10 (76.9)	3 (23.1)	1.50 (0.35–6.40)	
Running	−	230 (75.2)	76 (24.8)	1.00 (ref)	<0.05
	+	18 (62.1)	11 (37.9)	3.16 (1.14–8.75)	
Cycling	−	244 (74.2)	85 (25.8)	1.00 (ref)	NS
	+	4 (66.7)	2 (33.3)	1.02 (0.08–13.10)	
Duncing	−	237 (73.1)	87 (26.9)	1.00 (ref)	NS
	+	11 (100.0)	0 (0.0)	0.00 (0.00–Inf)	
Swimming	−	240 (74.1)	84 (25.9)	1.00 (ref)	NS
	+	8 (72.7)	3 (27.3)	0.63 (0.14–2.84)	
Muscle training	−	227 (73.7)	81 (26.3)	1.00 (ref)	NS
	+	21 (77.8)	6 (22.2)	1.05 (0.30–3.70)	
Stretching	−	193 (73.1)	71 (26.9)	1.00 (ref)	NS
	+	55 (77.5)	16 (22.5)	0.81 (0.37–1.78)	
Other sports	−	227 (73.5)	82 (26.5)	1.00 (ref)	NS
	+	21 (80.8)	5 (19.2)	0.33 (0.04–2.66)	

*Note*: Logistic regression analysis evaluated the effects of multiple variables, including walking‐related factors such as frequency, intensity, duration, and type, on depressed status in health‐conscious walkers. In the multivariate model, the odds ratios (ORs) were adjusted for the following: age, sex, alcohol drinking status, smoking status, working status, and regular exercise or sports other than walking (ball games, running, cycling, dancing, swimming, muscle training, stretching, others).

Abbreviations: CI, confidence interval; NS, not significant; OR, odds ratio; SD, standard deviation.

In non‐health‐conscious walkers (*n* = 589), logistic regression analysis to investigate factors affecting depressive status (CES‐D ≥ 16) showed that depressive status was significantly positively associated with walking <30 min per day (OR, 2.06; 95% CI, 1.19–3.56) (Table 3).

**Table 3:** Logistic regression analysis of the effects of walking‐related factors on depressive symptoms in non‐health‐conscious walkers.

Variables		Adjusted OR (95% CI)	*p* value
(Intercept)		0.16 (0.07–0.37)	<0.001
Age, mean (SD)		1.00 (0.99–1.02)	NS

Variables		Adjusted OR (95% CI)	*p* value
Sex	Male	1.00 (ref)	NS
	Female	1.43 (0.88–2.31)	
Alcohol drinking status	Drinking	1.00 (ref)	NS
	Non‐drinking	1.43 (0.88–2.31)	
Smoking status	Smoking	1.00 (ref)	NS
	Non‐smoking	0.67 (0.39–1.13)	
Working status	Employed	1.00 (ref)	NS
	Seeking work/unemployed	1.28 (0.73–2.24)	
Walking duration	≥60 min per day	1.00 (ref)	
	Between 30 and 60 min per day	1.19 (0.66–2.17)	NS
	<30 min per day	2.06 (1.19–3.56)	<0.05
*Type of regular exercises or sports other than walking*			
Ball games	−	1.00 (ref)	NS
	+	1.29 (0.40–4.12)	
Running	−	1.00 (ref)	NS
	+	0.50 (0.06–4.14)	
Cycling	−	1.00 (ref)	NS
	+	0.00 (0.00–Inf)	
Duncing	−	1.00 (ref)	NS
	+	1.38 (0.14–13.30)	
Swimming	−	1.00 (ref)	NS
	+	1.18 (0.12–11.60)	
Muscle training	−	1.00 (ref)	NS
	+	1.74 (0.70–4.33)	
Stretching	−	1.00 (ref)	NS
	+	0.41 (0.16–1.04)	
Other sports	−	1.00 (ref)	NS
	+	1.89 (0.68–5.24)	

*Note*: Logistic regression analysis evaluated the effects of multiple variables including walking‐related factors such as duration, on depressed status in non‐health consciousness walkers. In the multivariate model, the odds ratios (ORs) were adjusted for the following: age, sex, alcohol drinking status, smoking status, working status, and regular exercises or sports other than walking (ball sports, running, cycling, dancing, swimming, muscle training, stretch, other).

Abbreviations: CI, confidence interval; NS, not significant; OR, odds ratio; SD, standard deviation.

Logistic regression analysis evaluated the association between health‐conscious or non‐health‐conscious walking participants and depressive symptoms across age groups. The results shown Table 4, there were not associated with between health‐conscious or non‐health‐conscious walking participants and depressive symptoms among young age group (*N* = 179, CES‐D ≥ 16; *n* = 31), middle age group (*N *= 401, CES‐D ≥ 16; *n* = 86), and old age group (*N* = 337, CES‐D ≥ 16; *n* = 95).

**Table 4:** Association between health‐conscious or non‐health consciousness walking participants and depressive symptoms across age groups by logistic regression analysis.

Stratification	Variables		CES‐D score < 16	CES‐D score ≥ 16	Adjusted OR	*p* value
			Mean (SD)	Mean (SD)	(95% CI)	
Young age			(*N* = 148)	(*N* = 31)		
	(Intercept)				0.25 (0.17–0.37)	<0.001
	Health.conscious	−	124 (80.0)	31 (20.0)	1.00 (ref)	NS
		＋	24 (77.4)	7 (22.6)	1.17 (0.46–2.96)	
Middle age			(*N* = 315)	(*N* = 86)		
	(Intercept)				0.25 (0.19–0.34)	<0.001
	Health.conscious	−	217 (80.1)	54 (19.9)	1.00 (ref)	NS
		＋	98 (75.4)	32 (24.6)	1.31 (0.80–2.16)	
Old age			(*N* = 242)	(*N* = 95)		
	(Intercept)				0.41 (0.29–0.57)	<0.001
	Health.conscious	−	116 (71.2)	47 (28.8)	1.00 (ref)	NS
		＋	126 (72.4)	48 (27.6)	0.94 (0.59–1.51)	

*Note*: Logistic regression analysis evaluated the association between health‐conscious or non‐health conscious walking participants and depressive symptoms across age groups. Age groups were classified as young age (<40 years old), middle age group (between 40 and 65 years old), and old age group (≧65 years old).

Abbreviations: CI, confidence interval; NS, not significant; OR, odds ratio; SD, standard deviation.

The *χ* ^2^ test to characterize the subpopulation who paid attention to posture during walking showed that it contained significantly more participants who were elderly (*p* < 0.01), female (*p* < 0.01), and employed (*p* < 0.01) (Appendix Table S1).

## DISCUSSION

### Profile of health‐conscious and non‐health‐conscious walkers

There was a significant difference in walking duration between health‐conscious and non‐health‐conscious walkers. On residual analysis, health‐conscious walkers included a significantly lower proportion with walking duration >60 min per day and a significantly higher proportion with walking duration between 30 and 60 min per day. These paradoxical findings may be due to the fact that older people who retired tended to be more health‐conscious. Although the younger population tended to be non‐health‐conscious, the majority of them were employed and maintained social activities, and therefore, they needed to walk for various reasons other than maintaining health, including commuting.

Furthermore, there were significant differences between health‐conscious and non‐health‐conscious walkers in age and engaging in regular exercise or sports other than walking, such as running, cycling, dancing, swimming, and stretching. The proportion of subjects who regularly engaged in exercises other than walking, such as running, cycling, dancing, swimming, stretching, and other sports, was significantly higher in health‐conscious walkers than in non‐health‐conscious walkers. This may also be because elderly persons tend to exercises or sports other than walking as health consciousness and leisure activities. The results of the present study suggested that the majority of people who intended to walk for health tended to walk less than an hour. People who walked for various reasons other than health walked longer than people who intended to walk for health.

### Associations between walking parameters and depressive symptoms

Associations between depressive symptoms and walking parameters including FITT [walking parameter; walking of frequency, intensity, time (or duration), and walking style (brisk walking, posture, stride length, and arms swing)] were examined in health‐conscious walkers. On multiple logistic regression analysis, posture during walking showed a significant negative association with depressive symptoms. The other factors, including frequency, intensity, duration, and other styles of walking, were not significantly associated with depressive symptoms. The VIF ranged from 1.00 to 1.34 for frequency of walking, intensity of walking, walking duration, and style of walking, which suggested that multicollinearities among the walking parameters were ignorable. Remarkably, in those who paid attention to posture during walking, there were higher rates of elderly persons, females, and non‐employed persons than in those who did not pay attention to posture. Considering that being elderly,^39^ female,^40^ and unemployed^41^ can be risk factors for depressive symptoms, posture during walking may be associated with mental health, especially in such people. Previous studies have reported that posture during walking is associated with physical status, including low heart rate, low systolic blood pressure, multiple cardiovascular and neuropsychiatric morbidities, and mortality.^42^, ^43^, ^44^ These suggest that improvement of the physical condition by paying attention to the posture during walking may underlie the decrease in depressive symptoms. In addition, walking posture with a forward eye direction and large arm swing following the guidance of a therapist has been found to improve self‐efficacy,^45^ which supports the association between paying attention to posture during walking and mental health condition. The results of the present study showed that regular running, but not other exercises, was significantly positively associated with depressive symptoms. It was noteworthy that the current study suggested that a certain proportion of depressed people, defined as CES‐D scores ≥ 16 (the cut‐off score), run regularly. Whereas 7.3% of the non‐depressed population ran regularly, 12.7% of the depressed population ran regularly. Proportion of subjects who ran regularly was significantly different between non‐depressed and depressed populations as shown in Table 2 (*p* < 0.05). This finding was contrary to the previous findings supporting the benefit of running interventions for mental health problems, such as a previous study examining the association between a 2‐week running intervention and depressive symptoms in young adults, which reported that depressive symptoms decreased significantly after the running intervention.^46^ Several issues may need to be considered to explain the discrepancy. First, a U‐shaped quadratic equation model, that is, exceeding the optimal range of physical activity level, was associated with worsening depressive symptoms, may provide an explanation to the current findings. For example, whereas participants loaded with total physical activity for a week of 5300–9200 METs‐min (750 to 1300 kcal daily for a 60‐kg person) or physical activity of about 3–4 h per day maintained the best mental health wellbeing of all participants, those who were loaded with stronger intensity or longer duration of physical activities, which was potentially beyond the optimal range, and those who were loaded with less intensity or shorter duration of physical activities showed higher depressive symptoms.^47^, ^48^ The current study, along with previous findings, warns us that caution is needed because there is a certain proportion of depressed persons among those who exercise at an improper intensity and duration, and consideration of the proper intensity and proper duration of physical exercise may be beneficial for maintaining their mental health condition.

Second, there might have been reverse causality bias in the observation. Since regular running has been known to have a positive effect on depressive symptoms,^46^ and the community members in Shichigahama town have been exposed to the information regarding mental health promotion since the GEJE, including the benefit of proper exercise for maintaining mental health condition, a greater number of health‐conscious depressed people might have intended to engage in regular running than those with fewer depressive symptoms. Third, caution is needed when interpreting the data, since the sample sizes for evaluating subgroups of health‐conscious people engaging in various sports were relatively small (ball games: 13, cycling: 6, dancing: 11, swimming: 11, muscle training: 27, and stretching: 71), which might have caused type I and II errors.

In the current study, the frequency, intensity, and duration of walking were not associated with depressive symptoms, which was contrary to the results of previous studies.^49^, ^50^, ^51^, ^52^ For example, leisure time activity, including walking with a frequency of more than two times/week, intensity of light to somewhat hard level, or duration of 150 min/week, was associated with less depressive symptoms.^49^, ^50^, ^51^, ^52^ Notably, Shichigahama Town is located on a peninsula rising from the sea, and its landform consists of steep slopes. Therefore, participants in the current study may find it difficult to perform moderate‐intensity exercise, and some populations tend to engage in excessive intensity of physical activity during walking. Providing guidance on a practical way of maintaining moderate‐intensity walking adjusting to the environment of the area affected by a disaster may be needed to maintain mental well‐being.

As for non‐health‐conscious walkers, a walking duration <30 min showed a significant positive association with depressive symptoms, consistent with previous studies reporting a significant association between shorter walking duration and higher depressive symptoms in non‐post‐disaster settings^22^ and post‐disaster settings.^24^

Since age has been reported to be associated with depressive symptoms,^39^ we conducted stratified analyses by age group to explore whether the association between health‐conscious walking and depressive symptoms differed by age. However, due to the cross‐sectional design of this study, no definitive causal conclusions regarding the relationship between age and depressive symptoms can be drawn from our findings. Caution is needed that the small sample sizes in each age group might have reduced the statistical power of the analysis, making it difficult to detect potential effects.

The present study provides initial evidence to support negative association between paying attention to posture component of FITT and depressive symptoms. It is noteworthy that interventions such as Tai Chi and mindfulness training, which emphasize awareness of posture and bodily sensations, have been utilized in occupational therapy and demonstrated beneficial effects on depression.^53^, ^54^ Paying attention to posture during walking may have common mechanism with Tai Chi and mindfulness training underlying ameliorating effect on depressive state.

### Limitations

This study had several limitations. First, this was a cross‐sectional study based on a questionnaire survey conducted eight years after the GEJE; therefore, a causal relationship between the manner of walking and depressive symptoms was not determined. Second, FITT was evaluated based on self‐reported questionnaires and may therefore differ from actual FITT of the participants’ walking. Third, detailed information, including FITT regarding exercises other than walking, was not considered. Fourth, some of the excluded participants were unable to complete the questionnaire due to severe depressive symptoms, which may have introduced a selection bias. Fifth, the participants were residents in one of the administrative districts affected by the GEJE. The results might have been affected by factors specific to the community impacted by the natural disaster, and the findings need to be validated in other communities.

### Conclusion

The current study showed that paying attention to posture during walking had a significant negative association with depressive symptoms, suggesting that paying attention to posture while walking may be beneficial for mental health well‐being. In addition, walking time <30 min a day was positively associated with depressive symptoms in non‐health‐conscious walkers. These findings may help improve the mental health of communities affected by a disaster through an intervention promoting regular walking.

## AUTHOR CONTRIBUTIONS

Yusuke Utsumi and Hiroaki Tomita conceived the study. Yusuke Utsumi and Hiroaki Tomita conducted a major part of the data analysis and interpretation and drafted the manuscript. Yusuke Utsumi, Yumi Sugawara, and Hiroaki Tomita played major roles in survey management. Yumiko Hamaie, Atsushi Sakuma, Kazuho Tomimoto, Hiroshi Komatsu, Saya Kikuchi, and Yasuto Kunii contributed to data analyses and interpretation of the results. Moe Seto and Hitomi Usukura contributed to the management of the survey and interpretation of the data. Shinichi Kuriyama, Naoki Nakaya, and Atsushi Atsushi supervised the survey and data analysis and interpretation. All authors have read and agreed to submit the final draft.

## CONFLICT OF INTEREST STATEMENT

The authors declare no conflicts of interest.

## ETHICS APPROVAL STATEMENT

The research protocol was approved by the Ethics Committee of Tohoku University. The participants answered the questionnaires after providing informed consent.

## PATIENT CONSENT STATEMENT

All participants received written information about the study. Returning the completed questionnaire was considered as providing informed consent to participate.

## CLINICAL TRIAL REGISTRATION

N/A.

## Supporting information

APPENDIX Table S1.

## Data Availability

Detailed data are available upon request. In these cases, some information was excluded from the raw data to protect personal information.
