# Supplementary material for: Associations between depressive symptoms and frequency, intensity, duration, and style of walking in survivors of the Great East Japan Earthquake
Source: PCN Rep. 2025 Sep 14;4(3):e70178. doi: 10.1002/pcn5.70178 (PMC12433608; doi:10.1002/pcn5.70178)
Supplement: Supplementary file 1 — APPENDIX Table S1. [file PCN5-4-e70178-s001.pdf]

Table S1.Comparison of characteristics between two groups by whether they were aware of paying attention to posture while walking in health-conscious walkers

Factor	Group	Non-conscious（N＝131） Mean (SD)	Concsiousness（N=204） Mean (SD)	P-value
Age (mean, SD)		59.9 (15.5)	65.1 (15.6)	<0.01
**Factor**	**Group**	**Non-conscious（N＝131） Number (%)**	**Concsiousness（N=204） Number (%)**	**P-value**
Sex	Male	69 (47.9)	75 (52.1)	<0.01
	Female	62 (32.5)	129 (67.5)	
Alcohol drinking status	Drinking	54 (44.6)	67 (55.4)	NS
	Non-drinking	69 (36.5)	120 (63.5)	
Smoking status	Smoking	14 (46.7)	16 (53.3)	NS
	Non-smoking	108 (37.6)	179 (62.4)	
Working status	Employed	77 (48.4)	82 (51.6)	<0.01
	Seeking work / Unemployed	53 (30.8)	119 (69.2)	
Type of regular exercises or sports other than walking				
Ball games	-	125 (38.8)	197 (61.2)	NS
	+	6 (46.2)	7 (53.8)	
Running	-	117 (38.2)	189 (61.8)	NS
	+	14 (48.3)	15 (51.7)	
Cycling	-	130 (39.5)	199 (60.5)	NS
	+	1 (16.7)	5 (83.3)	
Duncing	-	128 (39.5)	196 (60.5)	NS
	+	3 (27.3)	8 (72.7)	
Swimming	-	126 (38.9)	198 (61.1)	NS
	+	5 (45.5)	6 (54.5)	
Muscle training	-	124 (40.3)	184 (59.7)	NS
	+	7 (25.9)	20 (74.1)	
Stretching	-	105 (39.8)	159 (60.2)	NS
	+	26 (36.6)	45 (63.4)	
Other sports	-	121 (39.2)	188 (60.8)	NS
	+	10 (38.5)	16 (61.5)	

Two groups, those who were and were not aware of “posture of walking”, were compared by age, sex, alcohol drinking, smoking, and regular exercise or sports other than walking (ball games, running, cycling, dancing, swimming, muscle training, stretching, others). The above parameters were compared between the two groups using the t-test and Chi-square test, as appropriate. SD; Standard deviation, NS; Not significant
